# Current Challenges and Perceived Future Priorities in Occupational Medicine: Perspectives From Occupational Health Professionals

**DOI:** 10.1002/hsr2.71515

**Published:** 2025-11-20

**Authors:** Khosro Sadeghniiat‐Haghighi, Nazanin Izadi, Nazila Heidari, Amirhossein Heidari, Fateme Seifollahzade

**Affiliations:** ^1^ Occupational Sleep Research Center, Baharloo Hospital Tehran University of Medical Sciences Tehran Iran; ^2^ Center for Research on Occupational Diseases (CROD) Tehran University of Medical Sciences (TUMS) Tehran Iran; ^3^ School of Medicine Iran University of Medical Sciences Tehran Iran; ^4^ Faculty of Medicine, Tehran Medical Sciences Islamic Azad University Tehran Iran

**Keywords:** challenges, qualifications, curriculum, Delphi study, occupational medicine

## Abstract

**Background and Aims:**

Advancements in knowledge and technology, along with the increasing complexity of occupational health requirements, make it essential to update the educational curriculum and standards in occupational medicine. This aims to improve worker health, reduce work‐related illnesses, and enhance the satisfaction of service recipients. This study was conducted to identify the current challenges in occupational medicine to support these goals in Iran.

**Methods:**

As part of this qualitative study, the Delphi method was utilized, with 20 occupational medicine specialists participating in the first phase, followed by 14 in the second and third phases. Initially, experts answered general questions on occupational medicine challenges. A second‐stage questionnaire with a Likert scale was created based on these responses. The comments were then reviewed, scored, and prioritized.

**Results:**

A total of 79 challenges were identified by the experts. Among these, the following issues were considered top priorities: the inability of occupational health specialists to prescribe medications and paraclinical tests under insurance coverage; the absence of a specialized occupational health committee within health departments and health offices to play a role in policy‐making, guideline development, and related activities; the lack of return‐to‐work examinations; the limited role of occupational health specialists in laws and guidelines as the primary authority for occupational health services. These services include return‐to‐work examinations, fitness‐for‐work evaluations, work exit examinations, case‐specific assessments, and compensation determinations.

**Conclusion:**

According to expert opinions, addressing the challenges in occupational medicine requires revising outdated labor and health regulations to reflect modern workplace risks. This is particularly concerning for mental health and remote work. Moreover, strict enforcement mechanisms should accompany these changes. Furthermore, integrating structured training in functional capacity evaluation into medical education can enhance specialization, professional standing, and workforce productivity.

## 1 Introduction

Occupational medicine is a medical specialty that investigates the complex interactions between work, the environment, and health [1]. This field is dedicated to identifying, evaluating, preventing, managing, and treating diseases and injuries that are related to the workplace. By applying knowledge from medical and health sciences, epidemiology, toxicology, ergonomics, safety, behavioral sciences, management, and associated laws, occupational medicine specialists work to maintain and promote the physical, mental, and social health of employees across various industries [2].

The scientific and academic study of occupational diseases began in the 18th century, specifically in 1700 A.D., when Bernardino Ramazzini published his seminal work, De Morbis Artificum Diatriba (“Diseases of Workers”) [3]. Alice Hamilton also focused on occupational toxicities, especially in workers exposed to lead [4]. At the beginning of the 20th century, hazardous exposures in the workplace increased. This led to a growing interest in and attention to occupational diseases and occupational medicine as a specialized field within the medical sciences. In 1904, the first occupational medicine clinics began to operate in Italy and other parts of Europe. In 1946, the American Academy of Occupational Medicine was founded in the United States [5]. In 1954, the instruction of occupational medicine as an academic specialty began independently in the United States.

Occupational health practices in Iran date back nearly a century, with early initiatives linked to labor protection laws in the 1940s, such as the Iranian Labor Law of 1946, which included introductory provisions for worker health and safety [6]. During the 1960s and 1970s, industrial growth led to the establishment of factory health units and the implementation of occupational hygiene practices in major industries, including oil, mining, and manufacturing [7]. However, formal academic training in occupational medicine began in 1995, when Tehran University of Medical Sciences launched the first structured 3‐year residency program [8]. Currently, five universities, including Tehran University of Medical Sciences in Iran, as well as Yazd, Mashhad, and Bandar Abbas, are recruiting and training residents for this specialized field.

More than 25 million workers in Iran are employed in various fields, including industry, agriculture, services, and administration [9]. Given the substantial costs associated with occupational diseases, including treatment, compensation, decreased productivity, and increased absenteeism, preventing these conditions is of utmost importance [10]. Therefore, it is essential to provide occupational health services and conduct periodic examinations for workers, as well as to ensure the training and development of specialists in this field.

Given the critical importance of establishing a qualification framework for graduates in each specialized field, and considering that the last revision of the occupational medicine curriculum occurred more than 15 years ago, it is imperative to update the curriculum. The evolving nature of this field, alongside medical advancements, particularly in the context of new educational methods such as virtual learning during the recent coronavirus pandemic, highlights the need for revision [11]. Additionally, the emergence of new occupational medicine groups in other universities, the expansion of industries and job roles, changes in exposure types and intensities, the rise of new diseases linked to occupational exposures, advancements in diagnostic, treatment, and prevention methods, and evolving work environment standards further necessitate the revision of both the occupational medicine curriculum and service standards.

It is necessary to evaluate the need for new laws, guidelines, and regulations or revisions to existing ones. Additionally, we must assess the requirement for new technologies and their localization, the need for curriculum revisions in educational programs, and the feasibility of approving more specialized courses in occupational medicine. Moreover, no prior studies in Iran have explored these challenges from the perspective of occupational health professionals. Therefore, this study offers a qualitative understanding of the key challenges and perceived future priorities in occupational medicine from the perspective of experienced occupational health professionals. To address the identified knowledge gap, this study aims to explore the current state and key challenges of occupational medicine in Iran, as perceived by specialists in the field of occupational medicine. By analyzing their insights, we identify critical themes, such as educational gaps, skill development needs, and regulatory shortcomings, that influence the practice and advancement of the field in Iran, thereby helping to inform future research and policy directions.

## 2 Methods

From November 2023 to August 2024, the Delphi study method (a type of qualitative study) was employed to benefit from experts' opinions. The protocol of the current study was reviewed and approved by the Ethics Committee of the Faculty of Medicine of Tehran University of Medical Science, Tehran, Iran (Approval code: IR.TUMS.MEDICINE.REC.1402.076). Moreover, informed consent was also obtained from all participants before enrollment. This method is commonly employed in uncertainty contexts to obtain expert consensus for informed decision‐making, especially in needs assessment, policy formulation, and priority setting [12]. The Delphi method offers several advantages, including engaging geographically diverse experts, maintaining anonymity to reduce bias, and combining qualitative insights with quantitative assessment in an iterative process [13, 14]. Our goal was not to develop formal guidelines, but to provide an informed and collectively validated overview of critical concerns as perceived by field professionals. Due to the consensus‐oriented nature of the Delphi process, we were able to move beyond individual viewpoints and highlight shared priorities, which enhanced the relevance and applicability of the findings [15]. Moreover, consensus in Delphi studies represents the convergence of expert opinion rather than an absolute truth, acknowledging the influence of diverse political, social, and disciplinary backgrounds [16, 17]. Notably, using this method, it is not possible to reach a 100% agreement, as people's political, social, economic, and scientific backgrounds differ [18]. The validity of the Delphi method is not determined by the number of participants but by the scientific expertise of those involved in the research [19]; thus, this method requires great accuracy when selecting the participants.

This study commenced with establishing a leading committee comprising the research team and several faculty members and occupational health specialists with academic, executive, and clinical experience. The committee defined the scope and boundaries of the project. The steering team developed the first‐round questionnaire following an initial investigation and data collection, as well as multiple brainstorming sessions. The questionnaire was designed to elicit expert opinion through open‐ended questions structured around four thematically grounded domains [1]: the current legislative and regulatory framework governing occupational health [2]; capacity‐building priorities and core competencies required for occupational health specialists [3]; the structural positioning of occupational health within the broader healthcare system, including intersectoral service delivery challenges; and [4] additional neglected or emerging issues not adequately addressed in existing frameworks. These domains were informed by an initial scoping review of international literature, including policy analyses and workforce development studies, and were further refined through consultations with subject matter experts. Collectively, they reflect critical dimensions commonly identified as determinants of occupational health system performance and serve as an organizing framework for the Delphi process. In the subsequent phase, the research team developed a comprehensive explanatory guide detailing the study's background, objectives, methodological framework, and standardized questionnaire instructions. This document is designed to enhance transparency, facilitate informed participation, and ensure consistent responses across all Delphi rounds. The Delphi method lacks clear and explicit rules and a formula for determining the sample size. The number of participants depends on the study's objectives, the scope of the issue, the quality of decisions needed, the research team's ability to manage the study, the internal and external validity, the timeline for data collection, and the resources available [20]. Delphi research participants usually include 5 to 20 people. The minimum number of participants depends on the research method's design. Some researchers note that as the number of participants increases, the answers become repetitive and no new information is added. In this study, 45 occupational health experts were initially invited, of whom 20 met the inclusion criteria and completed the first‐round questionnaire. The inclusion criteria for the study were a willingness to participate and meeting the scientific qualifications of the research population. In addition, non‐responsiveness, late submission, or incomplete questionnaires at any stage were excluded. In the next step, the first‐round questionnaire, accompanied by an explanatory guide, was distributed to participants either in person or electronically via email and secure online platforms. After returning the completed questionnaires, the research team reviewed the opinions expressed and omitted those that were similar. Following summarizing and editing the opinions (from a narrative point of view), the second‐round questionnaire was developed, incorporating a 7‐point Likert scale (ranging from ‐3 to +3) and accompanied by a revised guide (Completely agree = 3, Agree = 2, Partially agree = 1, I have no opinion = 0, Partially disagree = ‐1, Disagree = ‐2, Completely disagree = ‐3). Moreover, an explanatory text was prepared to guide participants on how to complete the revised questionnaire.

In the second round, 14 experts participated. In addition to scoring each item, participants were invited to provide qualitative feedback, namely, justifications for their responses and suggestions for solving each identified challenge. Of these, 12 out of 14 participants (86%) provided written suggestions or explanatory comments, contributing valuable qualitative insights alongside their quantitative ratings. During the third round, group feedback and justification were summarized, and participants were asked to revisit their positions on contentious items, based on the reasons for agreement or disagreement provided and the majority vote. Lastly, the challenges were prioritized based on the final scores. In detail, the maximum score that could be given to each opinion was three. If all these 14 people gave a score of three to an opinion, the total number of points would reach 42 (42 = 14 ×3). To determine consensus, a threshold of at least 70% of the maximum score was established. Those items that met or exceeded this threshold were classified as top‐priority challenges. In cases where items fell below the threshold, the expert panel revised them for clarity or omitted them from the final list.

## 3 Results

In the current study, we received 137 unique statements outlining perceived challenges in occupational medicine in the first round of the Delphi survey. A constant comparative method was used for systematic qualitative content analysis of these responses. We identified and consolidated similar themes across responses to minimize redundancy and enhance clarity. Items were considered duplicative when they addressed the same underlying issue with only minor wording or contextual framing differences, for example, multiple responses referencing limitations in prescribing authority under insurance coverage. A single representative statement was formulated in such cases, ensuring conceptual integrity while reducing redundancy. This synthesis process resulted in 79 distinct challenge items included in the second‐round Delphi questionnaire. Table 1 presents the complete ranking of all 79 challenges; however, several emerged as especially high‐priority, those receiving ≥ 70% agreement among participants, based on aggregated expert consensus. The top‐ranked challenges include the following: (1) Lack of prescribing authority and paraclinical access under insurance coverage for occupational medicine specialists (Score: 41/42). (2) Absence of a dedicated occupational health committee within health departments to support national policy‐making and guideline development (Score: 37/42). (3) Lack of mandatory return‐to‐work examinations, a critical component of post‐illness or post‐injury reintegration (Score: 36/42). (4) Limited legal recognition of occupational health specialists as the sole authority for conducting key assessments, including fitness‐for‐duty, exit evaluations, and compensation determinations (Score: 36/42).

**Table 1:** Ranking of occupational health challenges based on expert opinions.

Number	Challenges	Score (Out of 42)
1	The inability of occupational health specialists to prescribe medications and paraclinical tests (e.g., MRI, CT, etc.) under insurance coverage.	41
2	The absence of a specialized occupational health committee within health departments and health offices to play a role in policy‐making, guideline development, and related activities.	37
3	The lack of return‐to‐work examinations (necessitating the mandatory implementation of such examinations).	36
4	The limited role of occupational health specialists in laws and guidelines as the primary authority for occupational health services (e.g., return‐to‐work examinations, fitness‐for‐duty assessments, exit examinations, case‐specific evaluations, and compensation determinations).	36
5	The lack of effective supervisory tools and necessary enforcement measures to address violations in occupational health services (such as the absence of a responsible technical officer, lack of qualifications among service providers, failure to adhere to the standard limit of patient visits per center, etc.), along with inconsistent and discriminatory oversight practices.	34
6	Lack of education and the implementation of the practical concepts of functional ability.	33
7	Weakness in using the capacity of other specialized disciplines for training in interdisciplinary subjects.	33
8	Lack of consultation and referral from other specialists to an occupational medicine specialist regarding occupational diseases, determination of job restrictions, return to work, medical leave decisions, and related matters.	33
9	Unclear role of occupational medicine specialists as the only qualified physicians in the field of occupational health in “Labor Law”.	33
10	Lack of occupational health regulations and guidelines.	33
11	The absence of a comprehensive database and the ability to utilize registered data for analyzing and assessing the impact of occupational health examinations, risk assessment of exposures in the development and progression of occupational and nonoccupational diseases, and the identification of severe and chronic illnesses.	32
12	Granting authorization to general practitioners to conduct occupational health examinations which leads to the involvement of non‐specialists in occupational health services.	31
13	The lack of specialized training courses and workshops to provide certifications in fields such as disability issues, collaboration with forensic medicine, principles of human resource management, marketing, and financial resource management, HSE management, and courses related to artificial intelligence, informatics, and similar topics.	31
14	The lack of an efficient mechanism to provide the minimum number of referrals for occupational medicine specialists, especially newly graduated colleagues, and also to deal with the sinister phenomenon of tariff evasion.	31
15	Weakness in introducing occupational medicine services and their benefits to society.	31
16	Weakness in education and understanding of labor laws, social security, legal medicine, and the usual procedures for workers' complaints against the employer.	30
17	Failure to deal with diagnostic tests related to allergies and occupational sensitivities.	30
18	The need to employ occupational medicine specialists in the management system of occupational health (from the macro levels of the Ministry of Health to the lower levels of health vice‐chancellors and the occupational health office).	30
19	The low level of protection of people's personal data in files and the access of employers, and others to the complete contents of an individual's file.	30
20	The necessity of insurance coverage for sleep tests and treatment of sleep disorders, especially in safety‐critical jobs.	29
21	The neglect of issuing health cards for occupational groups within specialized occupational health services.	29
22	Lack of official employment of occupational medicine specialists in the judiciary.	29
23	Non‐mandatory pre‐employment and periodic examinations in all jobs.	29
24	Failure to understand malpractice from the point of view of the social security organizations, by specialists.	28
25	Lack of continuous and effective communication with insurance organizations, forensic medicine, the Ministry of Labor, etc. for, participation in commissions, cooperation in undertaking their plans and programs, developing guidelines, etc.	28
26	The lack of specifying the legal consequences of noncompliance with the conditions stated by specialists in the Labor Law.	28
27	Weakness in awareness of the latest version of the directives and instructions of the Ministry of Health, the Nuclear Energy Organization, etc.	28
28	The lack of involvement of occupational health specialists in the treatment domain, including the management of common diseases such as diabetes (DM) and hypertension (HTN), as well as the treatment of common musculoskeletal complaints, workplace‐related skin conditions, and other prevalent occupational health issues.	28
29	The lack of online case report meetings with the presence of professors, graduates, and residents.	27
30	Weakness in the laws related to preparing services in high‐risk situations (such as carcinogenic substances, etc.).	27
31	The disproportion between the malpractice from the point of view of the Social Security organization and the punishment determined by this organization (cancellation of the contract of the Social Security organization with the occupational medicine specialist causes a loss of billions for the specialist).	26
32	The necessity of a basic revision in the occupational medicine curriculum to include physical performance assessment tests and to provide both theoretical and practical training on these tests.	25
33	Unreasonably stringent regulations for obtaining a specialized center license.	25
34	Weakness in communication between educational groups and interested graduates.	25
35	Improper entry of specialists from other fields into the scope of duties of occupational medicine specialists by giving unnecessary medical leave and job restrictions to people.	25
36	Lack of mandatory occupational medicine consultants in large factories.	25
37	Weaknesses in training in interpreting MRI, CT scans, and commonly used radiographic imaging.	24
38	Deficiencies in training related to level III prevention with the aim of reducing the burden of disability and compromised ability (teaching rehabilitation measures, occupational therapy, and how to prescribe physiotherapy) to residents and specialists.	24
39	Failure to establish reference laboratories for biological monitoring.	24
40	Weakness in teaching the interpretation of EMG/NCV, supplementary audiometric, and visual tests.	23
41	The gap between regularly holding occupational medicine congresses and conferences with the view of scientific promotion and increasing the executive interactions of colleagues.	23
42	Weakness in training and introduction of jobs and occupational hazards during residency.	23
43	Weakness in the practical training of determining fitness for work.	23
44	The time‐consuming process of compensation payments to workers and the insufficient deterrent effect of compensation amounts for employers.	22
45	Authorized providers of occupational medicine services, the scope of their activities, and the level of services based on the skills and knowledge of the people are not specified in the rules and instructions.	22
46	Lack of Musculoskeletal Disorders Fellowship.	21
47	Lack of proper response to consultations by experts in other fields.	21
48	The failure to assign occupational health specialists as the primary authority in determining the extent of compensation for occupational injuries.	21
49	Lack of permanent and effective communication with the Iran Road Maintenance & Transportation Organization to identify problems in the health assessment of drivers.	21
50	Weakness in the training and entry of occupational medicine specialists in the fields of Health Impact Assessment, Environmental Health Product Stewardship, Medevac Procedure, and supervision of medical facilities of the project, Specific procedures for special hazards e.g., radioactivity, chemicals.	21
51	Failure to deal with the EMG/NCV test.	20
52	Inadequate qualification of the Social Security organization and the expert consultant of this organization to diagnose “medical malpractice” (due to conflict of interests).	19
53	Lack of entry of occupational medicine specialists in the field of rehabilitation and occupational therapy.	19
54	Lack of teaching of the general principles of methods of measurement, analysis, and interpretation of pollutant measurement in the work environment.	19
55	Weakness in teaching the diagnosis and treatment of musculoskeletal diseases.	19
56	Weakness in teaching the diagnosis and treatment of skin, lung, psychiatric, and sleep disorders.	18
57	The assignment of occupational health service implementation to health departments, which are responsible for supervising these services. In contrast, the involvement of the treatment department would be more technically appropriate, whereas health experts sometimes exceed their professional competencies in this field.	18
58	Lack of occupational therapy and corrective movements fellowship.	18
59	The lack of utilization of occupational health consultation services for assessing the psychological and physical fitness of individuals before choosing a field of study and determining their future career path in schools and higher education levels.	17
60	Lack of fellowship in occupational lung diseases.	17
61	The lack of attention to permit‐related medical examinations for specialized tasks within occupational health services.	17
62	The labor law only determines duties regarding occupational diseases, while most job restrictions are caused by nonoccupational diseases, and the labor law does not specify duties for the employer in this regard.	17
63	Lack of distinction between work‐caused and work‐related diseases in determining compensation in labor law.	17
64	Lack of training in designing targeted occupational health examinations for different occupations, which requires health risk assessment training.	17
65	Neglecting the issue of vaccination and passenger examinations in occupational medicine services.	16
66	The difference in approaches between insurance companies in different cities.	15
67	The lack of practical relevance of internal rotations and other rotations during residency training highlights the need to increase industry‐related rotations to enhance field experience.	15
68	Direct involvement of universities and the public sector in matters of job examinations and competition with the private sector.	15
69	Weakness in prioritizing occupational medicine research based on society's needs.	13
70	Absence of skin fellowship for occupational diseases.	13
71	The misalignment between occupational health service tariffs and the actual inflation rate, considering the rising costs of equipment, rent, staff salaries, and other expenses.	13
72	Failure to pay for procedures related to musculoskeletal disorders, such as joint injections, laser therapy, etc.	13
73	Absence of occupational disease research centers in the provinces.	12
74	The lack of cooperation from industries in providing access to research data.	12
75	Inadequate training in workplace alcohol and drug policies.	11
76	Not assigning all social security organization occupational examinations to occupational medicine centers.	9
77	Ineffectiveness of the current retraining courses for occupational medicine specialists.	9
78	Weakness in holding the article writings and data mining courses.	7
79	Absence of a toxicology fellowship.	2

Conversely, lower‐ranked challenges, such as the lack of toxicology fellowships or limited access to courses on academic writing and data mining, received minimal support and were considered less urgent by participants. As a third and final scoring round, the 14 participating experts assessed their previous responses based on aggregated group feedback and revised their scores on items where consensus was not reached. Based on the final scoring, future recommendations regarding educational reforms, workforce development, and policy‐level interventions in occupational health practice will be prioritized.

## 4 Discussion

This study was conducted to examine the current state of occupational medicine and its challenges from the perspective of occupational medicine specialists in Iran. Based on our results, the challenge of “the inability of occupational health specialists to prescribe medications and paraclinical tests under insurance coverage” has received the highest points. The independence and professional identity of occupational health specialists are undermined without this recognition. Until insurance organizations officially accept the authorization and stamp of occupational health specialists, the field will not achieve its rightful status as a recognized medical specialty. What is certain is that an occupational medicine specialist should have more insurance services than a general physician, especially since some paraclinical tests are a valuable tool for specialists in evaluating cases and are routinely used in completing occupational medicine cases. The idea of having more insurance services has its opponents. The influence of insurance organizations on the indication of tests, costs, and the manner in which tests are conducted is among the reasons for this opposition.

“The absence of a specialized occupational health committee within health departments and health offices to play a role in policy‐making, guideline development, and related activities” is the second most popular challenge from the point of view of experts. To effectively address problems within a given field, experts should be supported in applying their specialized knowledge to develop appropriate solutions. The allocation of a staff position for an occupational medicine specialist within the Ministry of Health and the Occupational Health Center is among the proposals put forward by experts to address this issue.

“The lack of return‐to‐work examinations” is another challenge raised by experts. Performing these evaluations is essential to supporting workers who cannot perform their duties due to illness, as they help prevent long‐term absences, reduce the risk of reinjury, and increase productivity. Due to a lack of standardized return‐to‐work protocols in Iran, workers and employers face uncertainty regarding fitness for duty and reintegration timelines. This has been documented in international contexts as well. To close the gap, relevant legislation must be enacted that mandates such evaluations. However, employers must also be proactively educated about their importance and how they can be effectively implemented in the workplace.

“The limited role of occupational health specialists in laws and guidelines as the primary authority for occupational health services (e.g., return‐to‐work examinations, fitness‐for‐duty assessments, exit examinations, case‐specific evaluations, and compensation determinations)” is another important challenge. Experts who participated in the current study stated that changes in legislation at the Council of Ministers level are needed to ensure clear decision‐making and to avoid personal or inconsistent actions by senior health officials.

“The lack of effective supervisory tools and necessary enforcement measures to address violations in occupational health services” are essential issues claimed by experts, which need amendment of the laws and the guarantee of their enforcement. Insights can also be gained from the approaches used by leading countries in occupational medicine to resolve similar conflicts and issues, provided that the solutions are appropriately adapted to the local context. Laws should be enforced equitably for both influential and marginalized individuals, as evidenced by studies [21, 22], which suggest that regulations are disproportionately applied to weaker individuals, while those with influence often find ways to circumvent them.

“Lack of education and then implementation of the practical concepts of the functional ability” is another serious challenge in the field of occupational medicine issues. In detail, the implementation of this concept in Iran remains limited due to the absence of clear national guidelines, inadequate training among occupational health professionals, and limited awareness or support from employers, all of which present significant challenges to its adoption. Assessing fitness for work without considering functional ability is an incomplete assessment. It may lead to reduced productivity, job loss, frustration, and an increased risk of physical and mental health disorders in the future. By specializing in occupational medicine services through the implementation of functional ability concepts, the entry of non‐specialists into the field of occupational health services will be prevented, thereby improving the field's status [23].

“Weakness in using the capacity of other specialized disciplines for training in interdisciplinary subjects” is another challenge highlighted. Occupational medicine requires a comprehensive understanding of multiple disciplines, as it involves assessing all bodily systems. Acquiring this knowledge necessitates interdisciplinary collaboration; however, factors such as conflicts of interest, lack of teamwork, and other barriers have thus far limited the effective utilization of this capacity.

“Lack of consultation and referral from other specialists to an occupational medicine specialist regarding occupational diseases, determination of job restrictions, return to work, medical leave decisions, and related matters” is another concern pointed out. To solve this problem, it is necessary to hold briefing sessions with scientific associations of other fields (e.g., it is necessary for experts of other fields to be aware of the damage they cause to large industries and small businesses by determining medical leave more than necessary, and sometimes even cause the loss of a person's job) and increase interdisciplinary scientific interactions [24]. Additionally, enhancing the specialized abilities of the field in domains such as impairment and functional ability is an effective solution to address this problem.

“Unclear role of occupational medicine specialists as the only qualified physicians in the field of occupational health in Labor Law” and “Lack of occupational health regulations and guidelines” are two other challenges in the field of occupational health laws [25]. Regarding the first challenge, experts noted that the lack of clarity regarding their role in labor law often leads to the legal invalidation of the Ministry of Health directives, which can serve the personal interests of profiteers.

Due to the methodological differences, classification approach, and novel perspective of our study, which is unique, no published research is available for direct comparison. In a 2018 study conducted at the international level, two key areas, “general principles of assessment and management of occupational hazards to health” and “assessment of disability and fitness for work,” were identified as primary qualifications for occupational medicine doctors [26]. Similarly, a 2016 study aimed to identify the competencies required of occupational medicine specialists worldwide, highlighting key priority areas, including general principles of occupational risk assessment and management for health purposes, disability and fitness‐for‐work evaluation, communication skills, and legal considerations [27]. In our study, there were similar challenges such as “The lack of specialized training courses and workshops to provide certifications in fields such as disability issues, collaboration with forensic medicine, principles of human resource management, etc”, “Weakness in education and understanding of labor laws, social security, legal medicine and the usual procedures for worker's complaint against the employer”, “Failure to understand malpractice from the point of view of the social security organization to specialists”, “Weakness in awareness of the latest version of the directives and instructions of the Ministry of Health, Nuclear Energy Organization, and…”, “Weakness in Introduction of jobs and occupational hazards, “Weakness in practical training of determining fitness for work”, “Lack of teaching of the general principles of methods of measurement, analysis, and interpretation of pollutant measurement in the work environment” and “gap in teaching how to purposefully design occupational medicine examinations for different occupations which necessitates training health risk assessment” were emphasized by experts, which shows the common concerns between our study and the aforementioned studies.

In another study carried out by Yungil Lee et al [28]. In 2015, residency programs in Korea were investigated and the residents were less satisfied with toxicology and environmental medicine training programs. In our study, probably due to the lack of required infrastructure, low demand for clients, and the existence of more important priorities in the field of occupational medicine issues, these cases have not been considered as the first priorities, and instead, emphasis has been placed on the training of functional ability concepts. In another study conducted across 347 European universities in 28 countries in 2024, the state of occupational medicine medical education was evaluated [29]. The results revealed that out of 347 universities surveyed, 53 medical schools from 19 countries responded, yielding a response rate of 15.3%. Occupational medicine was included in the curriculum in 89% of the responding institutions. The results demonstrated that several European universities provide insufficient education in the field of occupational medicine. Similarly, in our investigation, “Lack of education and then implementation of the practical concepts of the functional ability” and “Weakness in communication between educational groups and interested graduates” were mentioned by experts. Furthermore, a study conducted in the United Kingdom identified diminishing resources over recent decades, driven by social, economic, and political factors, as well as shifting trends in research policy, as significant challenges in occupational epidemiology [30]. The limitations, such as a lack of resources, difficulties accessing data and participants, and issues with workers' records, have harmed occupational medicine research. In comparison, our study identified “weakness in prioritizing occupational medicine research based on society's needs” and “lack of cooperation from industries in providing access to research data” as challenges, but these were considered less significant by our experts.

### 4.1 Strengths and Limitations

The Delphi method has several methodological limitations, including prolonged duration, participant fatigue across iterative rounds, potential attrition, ambiguity in expert selection criteria, lack of standardized consensus thresholds, and variability in sample size across studies [31]. It may also underestimate future‐oriented insights, as participants often prioritize present challenges, which can potentially reduce motivation and response accuracy. In this study, expert consensus on key challenges in occupational medicine was developed through an iterative process. In the first round, participants provided open‐ended responses, which were thematically analyzed and converted into structured items for a second‐round questionnaire. In this questionnaire, participants rated the items using a Likert scale, and items reaching the predefined consensus threshold ( ≥ 70% agreement) were retained, while others were revised or excluded based on expert panel review. Although the method offers a structured framework for consensus‐building, its validity relies heavily on participants' expertise and engagement; some participants declined to participate due to time constraints or perceived redundancy. In a few cases, personal or institutional interests appeared to influence responses, and the emphasis on majority opinions may have overlooked specialized minority viewpoints, meaning that consensus does not always represent the most accurate or expert perspective [15]. Additionally, while a larger sample size can enhance generalizability, it increases complexity and risks participant fatigue. In our study, the volume of feedback required for merging related items led to rating confusion and may have contributed to the generation of more generalized rather than concrete findings [32, 33, 34]. Another limitation of current study is that it exclusively reflects the perspectives of occupational health professionals; while their insights are essential to understanding the challenges within occupational medicine, the exclusion of other key stakeholders, such as workers, employers, policymakers, and academic researchers, may limit the comprehensiveness of the findings and their applicability to the broader societal context of evolving work environments and labor demands. Despite these limitations, the Delphi method proved effective in synthesizing expert input and establishing consensus‐based priorities, offering a valuable foundation for future research and policy initiatives in this evolving field.

## 5 Conclusion and Suggestions

According to the results of this study, the professional role of occupational medicine can be strengthened by integrating insurance services. Moreover, organizing, financing, and intersectoral coordination of occupational health services require legal reforms to overcome current challenges. To ensure successful implementation, experts should be involved in the development of these changes, and oversight should be adequate. Furthermore, the role of occupational medicine specialists as the primary authority in occupational health services must be redefined and strengthened within legal and regulatory frameworks. From an educational perspective, there is a critical need to incorporate training on functional ability to further specialize the field, elevate its status, improve employee performance, and ultimately enhance productivity. It is essential to note that the findings of the Delphi study represent expert opinions rather than absolute facts. Therefore, in policy‐making studies, considering the potential conflicts of interest and diverse intellectual perspectives, it is essential to incorporate input from all relevant stakeholders. Future research should examine the challenges of occupational medicine from the perspectives of employers, workers, faculty members, and occupational medicine residents to ensure a comprehensive and inclusive understanding of the field's issues.

## Author Contributions

**Khosro Sadeghniiat‐Haghighi:** writing – original draft, writing – review and editing, conceptualization, methodology, project administration, validation. **Nazanin Izadi:** conceptualization, writing – original draft, writing – review and editing, methodology, project administration, validation. **Nazila Heidari:** writing – original draft, writing – review and editing, investigation, validation. **Amirhossein Heidari:** writing – original draft, writing – review and editing, validation, investigation. **Fateme Seifollahzade:** data curation, supervision, investigation, validation, conceptualization, methodology, writing – review and editing.

## Disclosure

All authors have read and approved the final version of the manuscript (Fateme Seifollahzade), have full access to all of the data in this study, and take complete responsibility for the integrity of the data and the accuracy of the data analysis.

## Ethics Statement

The study protocol was reviewed and approved by the Ethics Committee of Tehran University of Medical Sciences, Tehran, Iran (Approval code: IR. TUMS. MEDICINE. REC.1402.076). All procedures followed were in accordance with the ethical standards of the responsible committee on human experimentation (institutional and national) and with the Helsinki Declaration of 1975, as revised in 2000.

## Consent to Participate

Informed consent was also obtained from all participants before enrollment.

## Conflicts of Interest

The authors declare no conflicts of interest.

## Transparency Statement

The corresponding author (Fateme Seifollahzade) affirms that this manuscript is an honest, accurate, and transparent account of the study being reported; that no important aspects of the study have been omitted; and that any discrepancies from the study as planned (and, if relevant, registered) have been explained.

## Data Availability

The data that support the findings of this study are available from the corresponding author (Fatemeh Seifollahzade) upon reasonable request.
